# Bioavailability of Heavy Metals in Soil: Impact on Microbial Biodegradation of Organic Compounds and Possible Improvement Strategies

**DOI:** 10.3390/ijms140510197

**Published:** 2013-05-15

**Authors:** Ademola O. Olaniran, Adhika Balgobind, Balakrishna Pillay

**Affiliations:** Department of Microbiology, School of Life Sciences, College of Agriculture, Engineering and Science, University of KwaZulu-Natal (Westville Campus), Private Bag X54001, Durban 4000, South Africa; E-Mails: adhikab@gmail.com (A.B.); pillayb1@ukzn.ac.za (B.P.)

**Keywords:** biodegradation, biosorption, chlorinated compounds, co-contamination, heavy metals

## Abstract

Co-contamination of the environment with toxic chlorinated organic and heavy metal pollutants is one of the major problems facing industrialized nations today. Heavy metals may inhibit biodegradation of chlorinated organics by interacting with enzymes directly involved in biodegradation or those involved in general metabolism. Predictions of metal toxicity effects on organic pollutant biodegradation in co-contaminated soil and water environments is difficult since heavy metals may be present in a variety of chemical and physical forms. Recent advances in bioremediation of co-contaminated environments have focussed on the use of metal-resistant bacteria (cell and gene bioaugmentation), treatment amendments, clay minerals and chelating agents to reduce bioavailable heavy metal concentrations. Phytoremediation has also shown promise as an emerging alternative clean-up technology for co-contaminated environments. However, despite various investigations, in both aerobic and anaerobic systems, demonstrating that metal toxicity hampers the biodegradation of the organic component, a paucity of information exists in this area of research. Therefore, in this review, we discuss the problems associated with the degradation of chlorinated organics in co-contaminated environments, owing to metal toxicity and shed light on possible improvement strategies for effective bioremediation of sites co-contaminated with chlorinated organic compounds and heavy metals.

## 1. Introduction

In tandem with rapid economic and industrial advancement, human activities have instigated widespread pollution of the natural global environment [1]. In recent years, the concern about the presence, disposition, and persistence of organic pollutants in the environment (air, soil, and water systems) has increased since most of the important classes of chemicals have been shown to be carcinogenic in experimental animals, thus posing a potential human health risk [2,3]. Chlorinated organic solvents are among the most widespread organic contaminants present in the groundwater and subsurface soils of many contaminated sites. The physicochemical properties of these chlorinated compounds, particularly as components of dense nonaqueous liquid phases (DNAPLs), make them difficult to remove once they have entered the subsurface and they remain among the most difficult contaminants to remediate in the environment [4]. Due to their latent toxicity to both wildlife and humans, production and use of several chlorinated organic chemicals have been completely banned in many countries around the world. Although several physico-chemical methods are available for treating these contaminants, each of these methods has inherent negative environmental impacts.

Bioremediation is an option that offers the possibility to destroy contaminants or render them innocuous using natural biological activity. Microbial degradation has been proposed as an efficient strategy for organic waste removal and it has distinct advantages over physico-chemical remediation methods; as it uses relatively low cost, low technology techniques, and may be carried out on site to achieve the complete degradation of organic pollutants without collateral destruction of the site material or its indigenous flora and fauna [5]. Also, biological processes and biodegradation of organic contaminants to innocuous end products (CO_2_, cell mass, water) minimizes the environmental impact and residual contamination [6,7]. However, anthropogenic chlorinated organic pollutants are now dispersed throughout the environment and can be highly recalcitrant to biodegradation processes found in most naturally occurring microorganisms. Also, the acquirement of biodegradative capabilities by indigenous microorganisms at contaminated sites through natural evolutionary processes often occur at an unacceptably slow rate, particularly when several biodegradation traits are required as in the case of co-contaminated sites.

Forty percent of hazardous waste sites on the Environmental Protection Agency’s (EPA) National Priority List (NPL) are co-contaminated with organic pollutants and heavy metals, and remediation of these sites pose a complex problem because of the mixed nature of contaminants. Co-contamination often causes a synergistic effect on cytotoxicity [8], and the two components often must be treated differently [9–11]. Such concerns have heightened the need for novel and advanced bioremediation techniques to effectively remove organic pollutants from a variety of co-contaminated environmental media including water, sediments and soil [1,12]. Metals most frequently found at United States EPA Superfund sites are divided into two categories namely; *cationic* metals (metallic elements whose forms in soil are positively charged cations) and *anionic* compounds (elements whose forms in soil are combined with oxygen and are negatively charged). The most common problem-causing cationic metals are mercury, cadmium, lead, nickel, copper, zinc and chromium, whereas the most common anionic compound is arsenic [13]. Common organic pollutants at these sites include petroleum, polycyclic aromatic hydrocarbons (PAHs), chlorinated solvents, herbicides and pesticides [9,14]. Few reports have focused on the adverse effects of heavy metals on biodegradation in co-contaminated environments, under both aerobic and anaerobic conditions. These effects include extended acclimation periods, reduced biodegradation rates and failure of the degradation of the target compound [15,16]. Complications of the effects of metal toxicity on organic pollutant biodegradation in co-contaminated soil and water environments stems from the fact that heavy metals may be present in a variety of chemical and physical forms, namely, soil adsorbed species, soluble complexed species and ionic solutes. Further impediments arise due to the impact of environmental conditions such as pH, redox potential of the water phase as well as soil properties, including ion exchange capacity, clay type and content and organic matter content, on the physical and chemical state of the metals [10].

To date, a paucity of information on the biodegradation of chlorinated organic pollutants in co-contaminated environments exists. Therefore, in this review, we discuss current problems associated with metal toxicity in co-contaminated environments, and shed light on possible improvement strategies for effective bioremediation of sites co-contaminated with chlorinated organic chemicals and heavy metals. This review specifically addresses: toxic metals and toxicity mechanisms to microorganisms; factors affecting the physical and chemical state of metals, which in turn influence metal speciation and bioavailability; measurement of bioavailable metal concentrations; influence of heavy metals on microbiological processes required for effective bioremediation; and improvement strategies aimed at increasing biodegradation in co-contaminated environments.

## 2. Metal Toxicity and Microbial Resistance Mechanisms

Metals play an integral role in the life processes of microorganisms. Some metals, such as calcium, chromium, cobalt, copper, iron, magnesium, manganese, nickel, potassium, sodium and zinc, serve as micronutrients and are used for redox-processes; to stabilize molecules through electrostatic interactions; as components of various enzymes; and for regulation of osmotic pressure [17]. Thus, metal ions may play important roles as “trace elements” in sophisticated biochemical reactions. Many other metals (e.g., silver, aluminium, cadmium, gold, lead and mercury), have no biological role and are nonessential and potentially toxic to microorganisms [17]. At higher concentrations these heavy metal ions form unspecific complex compounds within the cell, which leads to toxic effects, making them too dangerous for any physiological function. Toxicity of nonessential metals occurs through the displacement of essential metals from their native binding sites or through ligand interactions [17,18]. For example, Hg^2+^, Cd^2+^ and Ag^2+^ tend to bind to sulfhydryl (−SH) groups of enzymes essential for microbial metabolism, and thus inhibit the activity of sensitive enzymes [18]. To have a physiological or toxic effect, most metal ions have to enter the microbial cell. Many divalent heavy metal cations (e.g., Mn^2+^, Fe^2+^, Co^2+^, Ni^2+^, Cu^2+^ and Zn^2+^) are structurally very similar. Also, the structure of oxyanions such as chromate resembles that of sulfate, and the same is true for arsenate and phosphate. In such cases, these toxic metal cations may substitute for physiological essential cations within an enzyme rendering the enzyme non-functional. Thus, to be able to differentiate between structurally very similar metal ions, the microbial uptake systems have to be tightly regulated.

Most microorganisms have solved this problem by using two types of uptake mechanisms for heavy metal ions. One is fast, unspecific, constitutively expressed and driven by the chemiosmotic gradient across the cytoplasmic membrane of bacteria [18]. The second type of uptake system is inducible, has high substrate specificity, is slower, often uses ATP hydrolysis as the energy source and is only produced by the cell in times of need, starvation or a special metabolic situation [19]. Even though microorganisms have specific uptake systems, high concentrations of nonessential metals may be transported into the cell by a constitutively expressed unspecific system [18]. In addition, at high levels, both essential and nonessential metals can damage cell membranes; alter enzyme specificity; disrupt cellular functions; and damage the structure of DNA [17]. Also, concentrations of elevated levels of heavy metals impose oxidative stress on microorganisms [20]. As a consequence, some microorganisms have been forced to develop metal-ion homeostasis factors and metal-resistance determinants [17–19]. Because metal ions cannot be degraded or modified like toxic organic compounds, six possible metal resistance mechanisms exist: exclusion by permeability barrier; intra- and extra-cellular sequestration; active transport efflux pumps; enzymatic detoxification; and reduction in the sensitivity of cellular targets to metal ions [17–19,21–23]. One or more of these resistance mechanisms allows microorganisms to function in metal co-contaminated environments.

Heavy metals may inhibit pollutant biodegradation through the interaction with enzymes directly involved in biodegradation or those involved in general metabolism. The ionic form of the metal mediates inhibition of enzymes involved in pollutant degradation in heavy metal contaminated environments [24], indicating that metal toxicity is related to the concentration of bioavailable metal rather than the total or even total soluble metal concentration.

## 3. Metal Speciation and Bioavailability

Metals have been reported to inhibit organic pollutant biodegradation and affect degradation rates; however, widely varying degrees and patterns of inhibition have been reported, due to the lack of consistent methods to characterize metal toxicity. Most commonly, reports on metal inhibition of biodegradation have been related to the total concentration of a metal in the test system. However, this may not be the most appropriate measure as it has been well established that some metal species are more bioavailable than others [25–28]. Speciation can broadly be defined as the identification and quantification of the different, defined species, forms or phases in which an element occurs [29], while bioavailability is the fraction of the total amount of a metal in a specific environmental compartment that, within a given time span, is either available or can be made available for uptake by microorganisms from the direct surrounding of the organism. Metal speciation and the resulting bioavailability rather than total metal concentration determines the overall physiological and toxic effects of a metal on biological systems [27,28,30]. Unfortunately, few studies investigating the impact of metals on biodegradation have provided metal speciation and bioavailability data. Traditionally, the environmental risk caused by heavy metal pollution is determined by quantification of total metals after digestion with strong acids by using conventional analytical methods. Additionally, before using these analytical methods, environmental samples require laborious treatment to solubilize the metal ions from the solid matrix. However, conventional analytical methods are not able to distinguish between available and (potentially hazardous) and non-available (potentially non-hazardous) fractions of metals to biological systems. This is of particular interest with respect to solid environments, e.g., soils, because of the great adsorption capability of heavy metals to solid phase [31]. A range of different physicochemical processes in the soil or sediment system, governs the behaviour and fate of metals. In the water phase, the chemical form of a metal determines the biological availability and chemical reactivity (sorption/desorption, precipitation/dissolution) towards other components of a system. Often overlooked in metal toxicity studies is the importance of the pH of buffer used in microbiological media and the time at which a metal is added to a given medium [32,33]. Also, the level of inhibition will depend on the concentration and availability of the heavy metals, which are dependent on complex processes controlled by multiple factors including the nature of the metals and microbial species [14,34–38]. Some of the factors affecting metal speciation and bioavailability are discussed in Sections 3.1 and 3.2.

### 3.1. Binding Components and Physicochemical Characteristics in Medium and Soil

Many pH buffers are often present at higher concentrations than other medium components used in test systems [28,39,40], and are able to complex and precipitate metals, thus affecting metal speciation and bioavailability. For example, phosphate, probably the most common buffer used in the majority of studies [14,41–43], is well known for its ability to precipitate many metals and reduce their bioavailability [28]. Phosphate readily sequesters metals and reduces their bioavailability via the formation of insoluble metal phosphate species, even at neutral to mildly acidic pH values. In a predictive model of the concentrations of free ionic metals as a function of phosphate concentration in Bushnell Haas medium (Difco™, Sparks, MD) commonly used in biodegradation studies, at a relatively low phosphate concentration of 2.27 mM, 44% less free ionic cadmium exists in the medium containing phosphate than in the same medium not containing phosphate. Some metals are more sensitive to phosphate precipitation than others. Cobalt bioavailability was predicted to remain high (95%) in the free, ionic form as the phosphate concentration was raised to 15 mM, while free, ionic nickel was predicted to fall to 21% of its concentration in the medium free of phosphate [44].

The metal-complexing capabilities of some zwitterionic buffers used in microbiological media (e.g., HEPES (4-2-hydroxyethyl-1-piperazine-ethanesulfonic acid), MES (2-4-morpholino-ethanesulfonic acid), MOPS (3-*N*-morpholino-propansulfonic acid), PIPES (1,4-piperazine-bis-ethanesulfonic acid)) have been reported. However, metals tend to remain more bioavailable in the presence of zwitterionic buffers than in the presence of phosphate buffers, due to the fact that these buffers do not interact with metals as strongly as phosphate buffers. Mash *et al.* [45] indicated that MES and MOPS (50 mM) did not complex copper, while HEPES (35 mM) strongly complexed copper at pH 7.2. PIPES buffer has been reported to complex lead but not cadmium or copper [32,46].

Although available data suggests that Tris-base (2-amino-2-hydroxymethyl-1,3-propanediol) complexes many metals, limited quantitative complexation data has been gathered [47]. Hoffman *et al.* [9] reported that the degrees of inhibition of cadmium on naphthalene biodegradation by *Comamonas testosteroni* were different in each of three chemically-defined minimal salts medium (MSM) tested. Biodegradation was completely inhibited by 100 μM total cadmium in PIPES-buffered MSM and by 500 μM total cadmium in Tris- and PIPES-buffered MSM. Neither of the cadmium concentrations completely inhibited biodegradation in Bushnell-Haas medium. In addition, two unique patterns of cadmium inhibition were observed *i.e.*, a dose-dependent manner in Tris- and PIPES-buffered MSM, and a non-dose-dependent manner in Bushnell-Haas medium. In some media, metal speciation and bioavailability can change significantly prior to reaching equilibrium [33,47]. The impact of these changes on microbial growth has been previously described for a marine dinoflagellate (*Amphidinium carterae*) [33]. Specifically, the authors reported that copper was less inhibitory when added to an artificial seawater medium (HEPES-buffered) 24 h prior to cell inoculation than when added 2 h after cell inoculation. Hoffman *et al.* [9] also reported on the importance of time at which a metal is added to a medium, during naphthalene (NAPH) degradation, results indicated that 12 hours following the addition of cadmium, the Cd^2+^ concentration in the 500 μM total cadmium treatments decreased by 93%, falling below the concentrations measured in the 100 μM treatments. This change in cadmium bioavailability may provide a mechanistic explanation for some literature reports of non-dose-dependent patterns of metal inhibition [15,16,48,49].

One of the principal factors that influence the toxic effect of contaminants not yet satisfactorily studied is the variation in soil composition [50]. The physicochemical properties of soil can widely influence metal speciation and consequently, its mobility, bioavailability and toxicity [51]. Metals may be distributed among many components of soil solids and may be associated with them in different ways (ion exchange, adsorption, precipitation, complexation, or present in the structure of minerals). The dehydrogenase enzyme activity (DHA) assay was modified using resazurin (oxidoreduction dye) for toxicity analysis of combined mixtures of heavy metals and polycyclic aromatic hydrocarbons in soil [50]. The method was modified to take into account possible interaction of resazurin with complex soil matrix (adsorption on the soil components, influence of inorganic substances and humic acids). Results showed that the sensitivity of soils to contamination correlated reasonably well with organic matter, calcium and amorphous phase content, which were in good agreement with the investigations of other researchers [52–55]. In soil with relatively low organic matter content and significant quantity of amorphous phase, high sensitivity to contamination by heavy metals and persistence of organic pollutants were observed. Organic matter content has a strong influence on cation exchange capacity, buffer capacity as well as on the retention of heavy metals. Thus, metals present in organic soils contaminated with a combination of heavy metals are less mobile and less bioavailable than metals present in mineral soils [56]. Time and moisture effects are also important factors that need to be considered when interpreting short-term toxicity studies and when making predictions concerning possible long-term effects of heavy metals in the soil environment. Tom-Petersen *et al.* [57], observed a time-dependent reduction in total Cu in soil water extracts during a 220 day incubation; and noted that the retention of Cu in dry soil is much less pronounced than in soils with higher moisture content, and that alternating drying and rewetting did not enhance Cu retention as compared with soils kept under stable humid conditions.

### 3.2. pH and Redox Potential

At high pH, metals tend to form insoluble metal mineral phosphates and carbonates, whereas at low pH they tend to be found as free ionic species or as soluble organometals and are more bioavailable [44,47,58,59]. At acidic pH, more protons (H^+^) are available to saturate metal-binding sites; therefore metals are less likely to form insoluble precipitates with phosphates when the pH of the system is lowered because much of the phosphate has been protonated [28]. Under basic conditions, metal ions can replace protons to form other species, such as hydroxo-metal complexes. In some cases the hydroxo-metal complexes, such as those formed with cadmium, nickel, and zinc are soluble, while those formed with chromium and iron are insoluble. A small change in pH can decrease metal solubility and bioavailability by several orders of magnitude. For example, the solubility of cadmium was reduced 8.8-fold by an increase in pH from 6 to 7 in 1.3 mM phosphate. The dependence of metal bioavailability on pH varies between different metals. For example, a rapid decline in the concentrations of the free, ionic species of copper and zinc in minimal media was observed at pH values higher than 5, while the free, ionic form of cobalt remains prevalent until the pH value is higher than 8 [44].

Many studies have shown that pH mediates metal toxicity [60–62]. Increasing the pH reduces the toxicity of nickel to a variety of different organisms, including bacteria (*Serratia marcescens*), filamentous fungi (*Arthrobotrys conoides*, *Penicillium vermiculatum*, *Rhizopus stolonifer*), and yeast (*Cryptococcus terreus*) [63,64]. Under mildly basic conditions (pH 8.5), much of the nickel may not be bioavailable because it forms complexes with various ligands. It is also possible that the nickel was less toxic at a higher pH because some organisms may prefer basic environments to neutral or acidic environments. More commonly, increasing pH has been shown to increase the toxicity of zinc, copper, and uranium to certain algal species [60,65] and of cadmium to various bacteria (*B. subtilis*, *E. coli*, *M. luteus*, *S. bovis*), actinomycetes (*Micromonospora chalcea*, *Nocardia corallina*, *Streptomyces flavovirens*), and fungi (*Saccharomyces cerevisiae*, *Schizosaccharomyces octosporus*) [62,66]. Possible reasons for this occurrence may be due to cells being able to take up or adsorb more of the metal ions under high pH conditions [67,68]. Also, various functional groups associated with the membrane of microorganisms would be protonated under acidic conditions, reducing the electrostatic attraction between the metal cations and the membrane. A third possibility is that metals are removed from the cell more efficiently under acidic conditions by efflux pumps that are driven by the proton motive force [67]. Another possible explanation for increased toxicity at a higher pH is the formation of species that are more toxic, such as the hydroxo-metal species [69–71]. Maintaining a constant pH throughout the duration of an experiment is essential because of the dependency of metal speciation on pH. An appropriate buffering system is thus required to prevent large deviations in pH throughout an experiment. Studies examining the effect of metal toxicity on biodegradation usually use a buffer that has a neutral to mildly acidic pH range [10,14,16]. The operational pH range is determined by the pKa of the buffer, which is the pH at which half of the weak acid used for buffering is protonated. When the pH is beyond the operational range of a buffer, even small additions of acid, such as the excretion of acidic metabolic end products by microbes, may drastically change the pH and can result in unanticipated metal speciation events [28,47].

The redox potential (Eh) of an environment also influences metal speciation. Redox potential is established by oxidation-reduction reactions that tend to be relatively slow, particularly in soil environments. However, microbial activity can dramatically influence the rate and establishment of redox potential in soil. Reducing conditions (negative Eh) found in anaerobic media can result in metal precipitation with media components. For example, only 1% of the total zinc added to acetate enrichment anaerobic cultures was found to be in the aqueous phase [72]. A similar situation occurs in saturated soil systems in which carbonates and sulfides are present. Under these conditions, cationic metals such as Fe^2+^, Cd^2+^, and Pb^2+^ combine with sulfides to form nontoxic, insoluble sulfide deposits. The amount of sulfide present in soil systems can often be increased substantially by the activity of sulfate-reducing bacteria such as Desulfovibrio species, which reduce sulfate to sulfide. Kong [73] found that the soluble metal concentration in sediment slurries initially amended with 20 mg/L cadmium, copper, or chromium were below detection limits of 0.03–0.04 mg/L. Furthermore, at 100 mg/L added metal, only 1 mg/L cadmium and <0.12 mg/L copper and chromium were found in the aqueous phase. Under oxidizing conditions (positive Eh), metals are more likely to exist in their free ionic form and exhibit increased water solubility. In addition, pH may decrease slightly or even dramatically under oxidizing conditions. The classical example in this case is the formation of acid mine drainage, where sulfide and sulfur are oxidized by *Thiobacillus thiooxidans* to sulfuric acid, resulting in pH values ranging as low as ≤2, which further adds to increasing the solubility of metals.

## 4. Measurement of Bioavailable Metal Concentration

Measurement of bioavailable metal concentrations is a vital step towards determining the effects of metals on chlorinated organic pollutant biodegradation, since the environmental risk caused by heavy metal pollution is traditionally determined by quantification of total metals [44,74]. The development of sensitive, effective, and inexpensive methods that can efficiently monitor and determine the presence and amount of hazardous heavy metals is still at its infancy. Common analytical techniques used are ion chromatography, inductively coupled plasma, and polarography [75]. Heavy metals can also be determined with ion-selective electrodes. However, these methods are not able to distinguish between available and non-available fractions of metals to biological systems [76]. According to the standards, measurements and testing programme (SM&T) of the European Commission, the most suitable approach for certification of a soil sample to characterize the bioavailable fraction of metals was a single step procedure using EDTA and acetic acid. Thus, by convention, single step extraction procedures are mainly applied to soil samples to identify the bioavailable fraction, using a number of different reagents able to extract all, or part of the metals from soil [77]. Conventional methods are reaching the highest accuracy with low detection limits [78], but are expensive, time consuming, and require the use of highly trained personnel. However, the main drawback of chemical methods is the question of the transfer of the results obtained on non-biological systems to biological ones [74]. The current tendency to carry out field monitoring has driven the development of bioassays, biomarkers, and biosensors as new analytical tools able to provide fast, reliable, and sensitive measurements with lower cost; many of them aimed at on-site analysis. These tools have also gained much attention since they integrate all aspects of bioavailability, including; exposure, accumulation, and toxic effects at the receptor level [79].

### 4.1. Bacterial Biosensors

Recombinant bacterial sensors have been constructed and used for the determination of a specific metal. Ivask *et al.* [80] used recombinant luminescent bacterial sensors for the determination of the bioavailable fraction of cadmium, zinc, mercury, and chromium in soil. In their study, two bacterial recombinant heavy metal sensors were constructed based on two different receptor-reporter systems: one was inducible by Zn^2+^, Cd^2+^, and Hg^2+^, and the other by Cr^6+^ and Cr^3+^. The bacterial sensors used were not perfectly specific to one heavy metal, but responded to some “non-target” metals as well. In another example, the *mer-lux* gene fusion in *E. coli* was used to estimate bioavailable mercury in soil. The *mer*-promoter was activated when Hg^2+^, present in the cytoplasm of the biosensor bacterium, binds to *MerR*, resulting in transcription of the *lux* genes and subsequent light emission [76]. The luminescence-based bacterial sensor strains *Pseudomonas fluorescens* OS8 (pTPT11) and *Pseudomonas fluorescens* OS8 have also been used for mercury and arsenite detection, respectively, in soil extracts [81]. Other biosensors have been designed, based on bioengineered proteins. In these cases, the biosensor monitors conformational changes caused by the binding of the metal ion to the engineered protein [82]. Mercuric ion-binding regulatory proteins were used as the biological part of the biosensor [83]. The conformational change resulting from the binding of the metal ion to the protein caused a change in the capacitance, which was proportional to the concentration of the metal ions. An *Escherichia coli* bacterial biosensor was developed based on green fluorescent protein expression under the control of the *cadR* gene of *Pseudomonas aeruginosa* BC15. The constructed bacterial biosensor proved to be useful in determining the availability of heavy metals in soil and wastewater [84]. A review article on the design and development of enzyme-, DNA-, immuno- and whole-cell-based biosensors for the detection of heavy metal toxicity is available [85]

### 4.2. Immunoassays and Bioreporters

Numerous promising tools are being developed that use biological systems to quantify solution phase and bioavailable metal concentrations. One of the most attractive features of these tools is that they can be used in complex systems such as microbiological media and soil. Immunoassays, which can detect solution phase metal concentrations in the low μg/L range, have been developed for cadmium, lead, cobalt, nickel, and zinc. An immunoassay for mercury is commercially available [86,87]. An ELISA method was also developed for detection of mercury based on specific monoclonal antibodies against mercury-chelate. The concentrations of mercury in environmental water samples obtained by competitive indirect ELISA correlated well with graphite furnace atomic absorption spectrometry method for detecting mercury [88]. Artificially synthesized antigens of cadmium and lead were proven to have potential applications in immunoassays for the detection of these heavy metals [89]. An immunoassay using specific monoclonal antibodies was used to successfully detect cadmium, chromium, or lead under optimum conditions [90] Bioavailable metal fractions have also been measured using whole cell bioreporters, that produce a protein with measurable activity (e.g., LacZ) or light in response to bioavailable metal. Bioreporters for detection of mercury have been produced using both the lacZ system [91] and the luminescent lux system [92,93]. However, it should be emphasized that measurement of bioavailable metal can vary, depending on the metal resistance mechanisms of the bioreporter system used. A review of applications, advantages and limitations of immunoassays and bioreporters for metal detection is available [94].

### 4.3. Geochemical Modelling Software

In addition to biological-based approaches, geochemical modelling software, such as MINEQL+ (Environmental Research Software, Hallowell, ME) or MINTEQA2, can be employed to predict metal speciation patterns as a function of ionic strength and pH [95]. These programs take into account equilibrium constants for each ion in solution and accurately calculate the concentration of any metal species under specified conditions. The accuracy of programs such as MINEQL+ has been verified experimentally. For example a cadmium ion-selective electrode was used to determine the concentration of divalent cadmium ion in a minimal salts medium over the pH range from 4 to 7 [67]. The experimental concentrations were comparable to those predicted by the modeling software. These programs do not take into account all organic ligands present in complex media, so they are more comparable to experimental situations in minimal media. In complex media, it is difficult to calculate the concentrations of all components because the composition of complex ingredients (e.g., yeast extract, beef extract) differs slightly in every batch. At least three computational models have been developed to predict the impact of metals on organic biodegradation [14,41,96]. None of these models incorporates metal speciation and bioavailability. Thus, data generated by these models may only be meaningful for the medium or soil that was used to develop the model.

### 4.4. Diffusion-Based *in Situ* Techniques

Recently, a diffusion-based *in situ* technique known as diffusive gradients in thin-films (DGT) has been proposed for the measurement of labile metal species in soils [97]. This technique was developed on the premise that metal speciation in conventional methods of testing soil solutions may change during sampling and extraction. The potential use of DGT in assessing metal bioavailability was further demonstrated when Cu uptake by plants grown on a large number of soils was linearly correlated to DGT measurements while soil solution concentrations predicted a non-linear relationship [98]. Risk assessments of metal contaminated soils obviously require a comprehensible protocol for testing metal bioavailability and mobility. Such a test should ideally be applicable with minimum perturbation of the soil, without disrupting the equilibrium between solid and solution phases, and be sensitive to prevailing conditions [99].

## 5. Effect of Heavy Metals on Microbiological Processes Involved in the Biodegradation of Chlorinated Organic Compounds

The influence of heavy metals on microbial processes of individual strains and communities, such as respiration [100–105], luminescence [106–109], and nitrogen transformation [110,111] has been extensively reviewed. The impact of heavy metals on microorganisms has also been reviewed [54,112,113]. However, a dearth of information exist on the impacts of heavy metals on the biodegradation of chlorinated organic pollutants, thus additional studies that incorporate a variety of benchmark chlorinated organic chemicals and various manipulations of environmental factors that affect metal speciation and bioavailability are needed.

A few research efforts aimed at addressing the issue of co-contamination under aerobic conditions are listed in Table 1. The natural breakdown of 1,1,1-trichloro-2,2-bis(4-chlorophenyl)ethane (DDT), a persistent organochlorine pesticide, was inhibited in an arsenic co-contaminated soil resulting in an increased persistence of DDT in the soil environment studied [114]. The intrinsic breakdown of DDT to 1,1-dichloro-2,2-bis(4-chlorophenyl)ethane (DDD) in the presence of 2000 mg/kg arsenic resulted in a 50% reduction DDD concentration compared to background arsenic of 5 mg/kg. Thus, it was demonstrated that arsenic co-contamination has an inhibitory effect on the breakdown of DDT via DDD, and that as arsenic concentrations increased, the DDT: DDD and DDT: 1,1-dichloro-2,2-bis(4-chlorophenyl)ethylene (DDE) ratios also increased. The biodegradation of 2,4-dichloro-phenoxyacetic acid methyl ester (2,4-DME) in two microbial samples, namely, sediment and aufwuchs (floating mats of filamentous algae), from lake water, was inhibited in the presence of Cu, Hg, Zn, Cd and Cr [16]. Minimal inhibitory concentrations (MIC) varied according to the metals and the type of microbial sample tested and did not necessarily follow the toxicity patterns observed for the metal concentrations required for significant effects on *V*_max_ and *t*_1/2_. Zinc was the most toxic, in sediment samples, with an MIC of 0.006 mg total zinc/L, whereas mercury was most toxic in aufwuch samples, with an MIC of 0.002 mg total mercury/L. Inhibition of 2,4-dichlorophenoxyacetic acid (2,4-D) biodegradation in cadmium co-contaminated systems has also been reported [26]. Degradation by *Alcaligenes eutrophus* JMP134, a cadmium-sensitive 2, 4-degrader, occurred in the presence of up to 24 mg/L cadmium in mineral salts medium containing cadmium-resistant isolate and 0.060 mg/g cadmium in amended soil microcosms and field-scale soil bioreactors. Results also indicated that 10^4^ colony forming units (CFU) of *Alcaligenes eutrophus* JMP134/mL alone in the presence of > 3 mg/L cadmium in mineral salts medium did not degrade 2,4-D due to cadmium toxicity.

Reported metal concentrations that cause inhibition of anaerobic biodegradation of halogenated organic contaminants are listed in Table 2. The effects of Cd^2+^, Cu^2+^, Cr^6+^ and Hg^2+^ ions on dechlorination and biodegradation of 2-chlorophenol (2-CP) and 3-chlorobenzoate (3-CB), were found to be: increased acclimation periods, reduced dechlorination or biodegradation rates, and failure to dechlorinate or biodegrade the target compound [15]. It was suggested that the concentration at which these effects were observed was characteristic of the metal ion added, the target compound studied and the consortium being used. The biodegradation of 3-CB was shown to be most sensitive to Cd^2+^ and Cr^6+^ whereas 2-CP consortium was considered most sensitive to added Cd^2+^ and Cu^2+^. Since 2-CP and 3-CB were dechlorinated by distinct bacterial species, differences in metal sensitivity may have been specific to the dechlorinating species or the dechlorinating enzymes themselves. Interestingly, with Hg^2+^ at 1.0 to 2.0 ppm, 2-CP and 3-CP were biodegraded 133 to 154% faster than controls after an extended acclimation period, suggesting adaptation to Hg^2+^, perhaps via removal or transformation of mercury by mercury-resistant bacterial species.

## 6. Improvement Strategies for Increasing Biodegradation in Co-Contaminated Environments

Several approaches aimed at reducing the extent to which metals inhibit biodegradation of chlorinated organic compounds have focused specifically on lowering bioavailable metal concentrations and/or increasing metal resistance to facilitate biodegradation. Approaches include inoculation with metal-resistant microorganisms and the addition of treatment amendments that can reduce metal bioavailability. Phytoremediation has also shown promise as an emerging alternative cleanup technology for co-contaminated environments, and is currently under investigation. The various approaches are discussed in Sections 6.1 to 6.6.

### 6.1. Metal-Resistant Bacteria

In the case of co-contamination, the double stress imposed on the soil bacterial communities means that for effective *in situ* bioremediation of the organic contaminant, there must be metal-resistant microbes with appropriate degradative genes, or consortia of metal-resistant microbes with suitable catabolic capabilities [117]. Previously, bioaugmentation studies focused on the introduction of a microorganism that was both metal resistant and capable of organic degradation. However, under environmental conditions such an approach is often unsuccessful, probably due to the high energy requirements needed to maintain concurrent metal resistance and organic degradation. Recent approaches have demonstrated the use of a dual-bioaugmentation strategy and the role of cell bioaugmentation in the remediation of co-contaminated systems [26,117].

Unlike organics, metals cannot be degraded, and thus most biological heavy metal remediation approaches rely on the detoxification and immobilization of the metal both to reduce the biological toxicity and to retard metal transport [26]. Many factors influence the survival of organisms exposed to toxic levels of heavy metals, including lateral gene transfer (LGT) for the dissemination of resistance phenotypes throughout microbial communities [118,119] and changes in active-site residues that influence substrate specificity of metal homeostasis proteins [120]. Although metals are thought to inhibit the ability of microorganisms to degrade chlorinated organic pollutants, several microbial systems of resistance to metals are known to exist [18,121–123]; however there are only three possible mechanisms by which these systems operate. First, the accumulation of the particular ion can be diminished by efflux, an active extrusion of the heavy metal from the cell [19], which include: members of the resistance-nodulation-cell division (RND) protein family; export superfluous cations, cation diffusion facilitators (CDF family); serve as secondary cation filters in bacteria, P-type ATPases-basic defence against heavy metal cations and CHR protein family, NreB, CnrT. Second, cations, especially the “sulfur lovers”, can be separated into complex compounds by thiol-containing molecules. Third, some metal ions may be reduced to a less toxic oxidation state. A detailed review is available that describes modes of efflux-mediated heavy metal resistance in prokaryotes [124].

Most aerobic cells have a physiological redox range (−421 mV to +808 mV); therefore to be detoxified by reduction the redox potential of a given heavy metal should be within this range. Thus, heavy metals such as Hg^2+^ (+430 mV), arsenate (+139 mV), and Cu^2+^ (−268 mV) may be reduced by the cell, but Zn^2+^ (−1.18 V), Cd^2+^ (−824 mV), and Ni^2+^ (−678 mV) may not [18]. In the case of many metals, resistance and homeostasis involves a combination of two or three of the basic mechanisms mentioned. Dual bioaugmentation involving inoculation with both a metal-detoxifying and organic-degrading bacteria to facilitate organic degradation within a co-contaminated system was investigated [26]. Soil microcosms were constructed using uncontaminated sandy loam soil amended with 500 μg of 2,4-D/mL, and co-contaminated with 60 μg of cadmium to a final concentration of 60 μg/mL. This was followed by inoculation with *Ralstonia eutropha* JMP134, a 2,4-D degrader and *Pseudomonas* H1, a cadmium-resistant strain. Based on the results obtained, it was concluded that dual bioaugmentation with metal-detoxifying and organic-degrading microbial populations is effective for remediation of co-contaminated soil; however reducing bioavailable metal concentrations via sequestration prior to inoculation with the organic-degrading population will foster increased degradation. In another study, Pepper *et al.* [117] investigated the role of cell bioaugmentation and gene bioaugmentation in the remediation of co-contaminated soil. *Escherichia coli* D11, which contains plasmid pJP4 but does not have the chromosomal genes necessary for the transformation of 2-chloromaleylacetate to succinic acid, was used for gene bioaugmentation. The observation from this study suggests that the indigenous transconjugant population generated from *E. coli* D11 inoculation was better suited for subsequent 2,4-D degradation than the *R. eutropha* JMP134-inoculated soil, in which the presence of the 2,4-D degrading inoculant repressed transconjugant growth. However, the ultimate choice of cell or gene bioaugmentation will depend on the relative health potential of the recipient population, the degree of contamination, and the time frame available for remediation.

### 6.2. Treatment Amendments

Many studies have been carried out to evaluate the ability of different chemical amendments to immobilize heavy metals in polluted environments. These additives include organic materials, phosphate rocks, iron and manganese oxides and oxy-hydroxides and waste by-products rich in these oxides as well as alkaline agents such as lime and zeolite [125–133]. In general, these treatments prove to have an ameliorative effect on reducing the metal mobility or bioavailability. Cadmium sorption in three different minerals, vermiculite, zeolite and pumice was evaluated [134]. Results indicated that zeolite and vermiculite reduced soluble cadmium concentrations by 90% and the metal sorbed on zeolite was mainly present in the non-exchangeable form (70%) at the lowest cadmium concentration (30–120 μM). Furthermore, it was reported that the percentage of cadmium sorption in zeolite and vermiculite was independent of the initial cadmium concentration, and the mineral sorption capacity was closely dependent upon pH. In particular, cadmium adsorption on pumice was raised from 20 to 90% with an increase of pH from 4 to 7.5. Phosphate amendments, in particular, have been given much attention for the treatment of Pb-contaminated environments [125,127,130,135,136]. Despite the well-documented ability of treatment amendments to reduce metal mobility and bioavailability, not much attention has been directed towards determining microbial endpoints after the treatment of contaminated environments. Brown *et al.* [135] examined the effect of lime, phosphorus, red mud, cyclonic ashes, biosolids and water treatment residuals on the toxicity of cadmium, lead and zinc in an international inter-laboratory study. Each participating laboratory selected a common soil material, from mine wastes and common treatments. Nitrogen (N) transformation and a measure of the total soil microbial biomass were chosen as microbial endpoints. The N transformation test was designed to measure nitrate formation in soils after the addition of an organic substrate. The formation of nitrate is an indicator of microorganisms degrading the C-N bonds in the organic substrate and recycling nutrients within the soil. Of the amendments tested by the participating laboratories, P added as either triple sugar phosphate or H_3_PO_4_ appeared to be the most effective. Phosphorus addition to the soil resulted in reduced soil solution and extractable metals, reduced bioavailability of soil Pb, and increased microbial activity based on the two measures. These promising results suggests that the use of treatment amendments may be an effective means to increase chlorinated organic pollutant biodegradation in the presence of toxic levels of heavy metals, however there were significant differences in efficacy within categories of amendments.

### 6.3. Clay Minerals

The use of clay minerals to reduce metal bioavailability and resulting toxicity in groundwater and sub-soils has been successful for the remediation of heavy metal polluted environments [10,137,138]. Clays differ in chemical and physical properties but show, in general, a comparatively high ion exchange capacity of 5 to >50 meq/100 g clay (montmorillonite > illite > kaolinite) and charged substances attach easily to clay particles. Sorption of heavy metals on clays has been studied for montmorillonite [139], illite [140], kaolinite [141,142], bentonite and vermiculite [134,143]. These clays are chosen to avoid pollutant release into the environment owing to their high specific surface areas, low cost and ubiquitous presence in most soils [144]. In particular, the evaluation of the total capacity of Na-montmorillonite shows that this clay is a good sorbent towards a variety of metals [145] and shows generally higher sorption of heavy metals than kaolinite [139]. This clay mineral adsorbs heavy metals either via cation exchange in the inter layers resulting from the interactions between ions and negative permanent charge or through formation of inner-sphere complexes through Si-O^−^ and Al-O^−^ groups at the clay particle edges [146–148]. It has been reported that the adsorption of metal ions on Na-montmorillonite decreases with decreasing pH and is also influenced by the presence of ligands [145]. At low pH values (2.5–3.5), the hydrogen ion competes with the heavy metals towards the superficial sites and, moreover, the Si-O^−^ and Al-O^−^ groups are less deprotonated and they form complexes with bivalent and trivalent ions in solution with greater difficulty. This effect was particularly evident for Cu^2+^ (as aqua ion [Cu (H_2_O) _6_]^2+^), which has a distorted geometry and for Pb^2+^ and Cd^2+^ that have a lower electrostatic attraction *versus* the clay because of their lower charge density. For these reasons, the adsorption of these ions is unsupported by cation exchange mechanism and, hence, they are more influenced by pH variations. Therefore, the pH effect was different on each metal and at pH ≤ 3.5, the studied metals were adsorbed in increasing amounts in the following order: Cu^2+^ < Pb^2+^ < Cd^2+^ < Zn^2+^ ≤ Mn^2+^ ≈ Cr^3+^ ≈ Ni^2+^. The effect of the ligand was a function of the ligand and metal considered, but the formation of metal-ligand complexes in solution altogether hinders the adsorption of the metal ions on the clay. In this case, the metal adsorption increased in the following order: Cr^3+^ < Cu^2+^ < Ni^2+^ < Zn^2+^ ≤ Cd^2+^ ≤ Pb^2+^ ≤ Mn^2+^. The results evidenced that the sorption capability of Na-montmorillonite towards each metal ion examined was different in the various conditions, and is a function of both pH and of the ligand present in solution. It is therefore necessary, to consider both these factors to study a real soil/solution system and effectively predict the fate of heavy metals in the environment. From the results it was evident that the total capacity of Na-montmorillonite towards the investigated metals increases in order: Pb^2+^ = Cd^2+^ < Cu^2+^ < Zn^2+^ < Mn^2+^ < Ni^2+^ < Cr^3+^. In a similar study, the LC_50_ of *Spumella* sp in solution systems contaminated with Cd^2+^ decreased by 71% and 64% in the presence of clay and silicate beads, respectively [137]. A recent review described the adsorption of heavy metals onto two common clay materials, namely kaolinite and montmorillonite [149]. The adsorption of Ni(II) ions on treated, spent, activated clay was found to increase with increasing shaking time and temperature and that about 99.9% removal was obtained at the optimum pH of 11 [150].

### 6.4. Chelating Agents

Chelating agents increase metals diffusion in the soil solution and keep them in plant available forms by forming large, less reactive ions, by increasing the concentration of these larger chelated ions in solution, and by decreasing the ability of the free ions to react with the soil. Chelating agents offer great promise for assessing readily available micronutrient cations in soils [77]. These agents adhere with free metal ions in solution forming soluble complexes and thereby reduce the activities of the free metal ions in solution. In response, metal ions desorb from soil particles or dissolve from labile solid phases to replenish the free metal ions in solution. Chelating agents, such as ethylenediamine-tetraacetic acid (EDTA), have been employed to reduce metal toxicity to organic-degrading microorganisms. EDTA was shown to reduce the toxicity of cadmium to *Chlorella* sp. [151], of nickel to algae [152] and of copper to bacteria and algae [153]. However, the toxicity of EDTA to many microorganisms and its limited biodegradability reduce its suitability for application to the bioremediation of co-contaminated environments [154,155]. Removal of copper and nickel in soil was evaluated by the addition of chelating agents, chitosan and ethylenediamine tetraacetic acid, using sodium citrate as the reference compound. The experiments showed that the extraction ability for copper and nickel from the contaminated soil decreased as follows: chitosan > EDTA > sodium citrate [156]. Raw ROU2 clay was proven to efficiently remove copper(II), lead(II) and chromium(III) from aqueous phases, mainly due to the high pH values obtained by the presence of carbonates. The development of several surfactant-modified clay complexes to reduce metal toxicity has therefore sparked greater interest [157]. Potassium butyl dithiophosphate (PBD) has potential as a chelating agent to remove heavy metal. Experimental results showed that PBD could be used to treat acidic Cd^2+^ wastewater with a removal rate that could reach over 99% [158].

The potential application of surfactant-modified clay adsorbents in mixed-waste biotreatment, in which toxic organics and heavy metals coexist, has been demonstrated [159]. In this study, the toxicity of cadmium to *Pseudomonas putida* was greatly reduced by the addition of a surfactant modified-clay complex and a commercially available chelating resin (Chelex 100; Biorad, Hercules, CA, USA) during the biodegradation of NAPH. Surfactant modified-clay complexes were prepared through a simple surface modification method of grafting metal-chelating ligands in order to impart a higher metal capturing capacity and selectivity to the base clays. In general, the modified-clay complex is prepared by a simple two-step method involving adsorption of a cationic surfactant, such as cetyl benzyl dimethyl ammonium (CBDA), and then anchoring of various metal-complexing ligands, such as palmitic acid (PA), through hydrophobic interactions to form a stable mixed bilayer of CBDA and PA on the clay surface. NAPH biodegradation occurred at higher cadmium concentrations in the presence of either chelex 100 or the modified clays than in the controls containing either no clay or unmodified clay.

### 6.5. Biosurfactants

Biosurfactants are amphiphilic compounds which can reduce surface and interfacial tensions by accumulating at the interface of immiscible fluids and increase the solubility, mobility, bioavailability and subsequent biodegradation of hydrophobic or less soluble organic compounds [160], such as polychlorinated biphenyls [161]. Biosurfactants are produced extracellularly or as part of the cell membrane by bacteria, yeasts and fungi, from various substrates including sugars, alkanes, oils and waste [162,163]. It has been proven that specific interactions occurring between rhamnolipids and chlorinated phenols decreases their toxicity and therefore stimulates biodegradation of diesel fuel hydrocarbons by a consortium of degraders [164]. Many studies of biosurfactant-enhanced bioremediation have employed well-characterized biosurfactants such as *Pseudomonas aeruginosa* rhamnolipids [162,165], *Candida apicola* sophorose lipids [166], *Rhodococcus erythropolis* trehalose dimycolipids [167] and *Bacillus subtilis* surfactin [168]. In addition to biodegradation of hydrocarbon degradation biosurfactants can be used to remove heavy metals. Chromium removal and its association with rhamnolipid production in *Pseudomonas* spp. were investigated [169]. Results indicate that there was a positive correlation between chromium removal and rhamnolipid production. A previous study revealed that triplicate washing of copper, cadmium and soil with the biosurfactant saponin increased both efficiency of metal removal and percentage content of their stable forms [170].

Of importance is the advantage of such compounds at co-contaminated sites, since microorganisms have long been shown to produce potent surface-active compounds that enhance the rate of degradation by emulsification or solubilization of the hydrophobic hydrocarbon [171]. Exploiting this property, Berg *et al.* [172] described the potential use of biosurfactant produced by a *P. aeruginosa* UG2 to significantly increase the solubility and dissolution of hexachlorobiphenyl into the aqueous phase. Furthermore, biosurfactants may also enhance the desorption of heavy metals from soils via 2 approaches: firstly, complexation of the free form of the metal residing in solution, thus decreasing the solution phase activity of the metal and promoting desorption according to Le Chatelier’s principle. Secondly, direct contact of biosurfactant to sorbed metal at solid solution interface under conditions of reduced interfacial tension, which allows biosurfactants to accumulate at solid solution interface [173]. A review article discusses the use of biosurfactants as an eco-friendly remediation method of environments contaminated with organic and inorganic contaminants such as hydrocarbons and metals [174]. Considerable work has been done on rhamnolipid biosurfactant produced by various *P. aeruginosa* strains capable of selectively complexing several cationic metal species, thus increasing the bioavailability of substrates with limited aqueous solubilities [175,176], and increasing cell surface hydrophobicity [177]. Research has shown that rhamnolipids complex preferentially with toxic metals such as Cd and Pb than with normal soil metal cations such as Ca and Mg, for which they have a much lower affinity [16].

Clay and iron oxide contents affect the efficiency of the biosurfactants but this has not been adequately researched. Anionic biosurfactants were found to be more effective where metals are the agents to be sequestered. Surfactin, rhamnolipid, and sophorolipids, all anionic biosurfactants, were able to remove copper and zinc from a hydrocarbon-contaminated soil [178]. In co-contaminated soil, the biosurfactants can potentially be produced *in situ* using the organic contaminants as substrates, subsequently leading to remediation of both contaminants, thus greatly reducing the remediation cost [179]. The efficiency of biosurfactants for stimulating biodegradation of contaminants is uncertain given the specificity observed between biosurfactant and organism. Addition of biosurfactant can stimulate some organisms but also can inhibit some microorganisms; a strategy suitable for effective remediation would therefore be to stimulate biosurfactants produced by indigenous population or use commercial biosurfactants produced by organisms found to be present at the contaminated site. Furthermore, delivery of a biosurfactant into co-contaminated sites for *in situ* treatment may be more environmentally compatible and more economical than using modified clay complexes or metal chelators such as EDTA [11].

### 6.6. Phytoremediation

*In situ* bioremediation is gaining momentum as a low-cost and effective method for restoration and remediation of many contaminated sites. In particular, the use of plants for rehabilitation of heavy metal contaminated environments is an emerging area of interest because it provides an ecologically sound and safe method for restoration and remediation [180,181]. A recent review provides an updated description of information existing with respect to metal tolerance and accumulation mechanisms in plants, as well as on the environmental and genetic factors affecting heavy metal uptake [182]. Plant improvement for enhanced phytoextraction and limitations on phytoremediation has also been discussed [183]. Although phytoremediation is a slow process, improvement of efficiency and thus increased stabilization or removal of heavy metals from soils is an important goal [184], especially in the case of co-contamination. The mechanisms used by plants to facilitate remediation include: phytoextraction, phytopumping, phytostabilization, phytotransformation/degradation, phytovolatilization, and rhizodegradation [185,186]. The biomass production of a few hyperaccumulator plants has been judged sufficient for phytoremediation; for example, the brake fern *Pteris vittata* accumulated up to 7500 μg/g As on a contaminated site [187], without showing toxicity symptoms. In soil co-contaminated with pentaphlorophenol (PCP) [initial concentration of 50 mg/kg] and copper, *Lolium perenne* L (ryegrass) and *Raphanus sativus* (radish) were observed to grow better with the increase in soil Cu level (0, 150, 300 mg/kg). This implied that combinations of inorganic and organic pollutants sometimes exerted antagonistic effects on plant cytotoxicity [8]. The lower level of PCP dissipation in copper co-contaminated soil has been attributed to the lowered degrading activity of microorganisms and the reduced mass flow [8]. The Thlaspi family have been shown to hyperaccumulate metals, with 23 species, 10 species and 3 species known to hyperaccumulate Ni, Zn and Cd, respectively, while only one species hyperaccumulate Pb. Of these species, *T. caerulescens* is able to grow in serpentine soils, which contain high levels of heavy metals, including Zn, Co, Pb, Cr, Cd and Ni and able to absorb up to 30.000 and 1000 mg/kg Zn and Cd, respectively in their shoot, without affecting its growth [188]. The three hallmarks that differentiate hyperaccumulators from related non-hyperaccumulator taxa are: a much greater capability to tolerate and take up heavy metals from the soil; a faster and effective root-to-shoot translocation of metals; and a much greater ability to detoxify and sequester huge amounts of heavy metals in the leaves [182]. The potential of phytoremediaion of soil co-contaminated with heavy metals and benzo[a]pyrene (B[a]P) with *Tagetes patula* was explored. The results from this study concluded that *T. patula* could be effectively used for phytoremediation of B[a]P and B[a]P–Cd contaminated soils [189]. The crop *Ricinus communis* has also been proven to have immense potential for eradicating dichlorodiphenyltrichloroethane (DDT) and cadmium from contaminated soils due to its fast growth, high biomass, strong absorption and accumulation of the co-contaminants [190]. Plant-available metals include species that are readily soluble or exchangeable, while metals that are more strongly adsorbed/bound to organic matter or co-precipitated with oxides are generally not available for plant uptake [10,44]. Natural chelating agents (organic acids such as citric and acetic) released by plant roots make the ions of both nutrients and contaminants more mobile in the soil. Plants can usually break the chelation bond, take up the metal, and release the chelant back in the soil solution [44]. Since most organic-degrading microorganisms are sensitive to the toxic effects of heavy metals, a successful strategy to address this mixed-waste situation requires the use of microorganisms that will survive and thrive in soil polluted with heavy metals. In addition to plants, plant associated microbes can play a vital role in phytoremediation and this is fully discussed by Rajkumar *et al.* [191]. The connections between a hyperaccumulating plant (*Sedum alfredii*) and bacteria (*Burkholderia cepacia*) on soil co-contaminated with heavy metal and phenanthrene was described. Inoculation of bacteria has been indicated to enhance metal tolerance of *S. alfredii* and increased its phytoextraction efficiency for Cd and Pb. The bacterium used in this study also increased the removal of phenanthrene [192]. Another study demonstrated that the planting of *S. alfredii* in addition to inoculation of a DDT degrading strain DDT-1 to potted soils could increase the root biomass of *S. alfredii* [193]. This resulted in effective phytoextraction of Cd from co-contaminated soils as well as enhanced rhizodegradation of DDT in the soil. The results of these studies indicate that the application of plants together with microbes appears to be a promising approach for the bioremediation of sites co-contaminated with heavy metals and organic compounds.

An attractive feature of using rhizoremediation in such a situation is the flexibility of utilizing different engineered rhizobacteria to remediate mixed-waste co-contaminated soil [194]. The rhizosphere bacterial community can be specifically engineered to target various pollutants at co-contaminated sites to provide a modified rhizoremediation system [181]. Specific biodegradation genes and plant species can be selected in accordance to the pollutants present and plant growth conditions at the toxic sites [181].

## 7. Conclusions

Determination of the effects of heavy metals on the biodegradation of chlorinated organic pollutants often yields conflicting results, both in aerobic and anaerobic systems. Such inconsistencies on ecotoxicity can be attributed to overgeneralization of the outcomes from short-term laboratory studies that focus on a single parameter under controlled conditions. To better characterize the inhibitory effect of toxic metals on biodegradation, additional studies are needed that incorporate a variety of benchmark chlorinated organic chemicals and various manipulations of environmental factors. The bioavailable fraction of heavy metals, the key factor in risk assessment analysis, varies with speciation, nature of applied metal salt, medium components/soil type, pH, redox potential and other environmental variables. Thus, no single parameter can be adequate to generalize heavy metal toxicity, necessitating the need for a battery of assays with sensitive, reliable and ecologically relevant biological tools to provide an understanding of environmental situations in which biodegradation is unexpectedly low, such as in co-contaminated environments. It will also enable regulatory guidelines to be established for co-disposing of chlorinated organics with toxic heavy metals.

## Figures and Tables

**Table 1:** Reported metal concentrations that cause inhibition of biodegradation of chlorinated organic contaminants under aerobic conditions.

Metal	Organic	Lowest metal concentration	Microbe(s) studied	Environment	pH	Reference
As^3+^	DDT	5 mg/kg a	Indigenous community	Former co-contaminated soil	NR	[114]
Cu^2+^	2,4-DME	0.027 mg/L a	Indigenous community	Aufwuchs (microcosm)	5.0	[16]
Cu^2+^	2,4-DME	0.076 mg/L a	Indigenous community	Sediment (microcosm)	6.1	[16]
Cu^2+^	4-CP, 3-CB, 2,4-D	<14.3–71.6 mg/L a,b	*Alcaligenes* sp., *Pseudomonas* sp., *Moraxella* sp.	Tris-buffered minimal medium plates	7.0	[115]
Cd^2+^	2,4-D	0.060 mg/g a	*Alcaligenes eutrophus* JMP134	Soil microcosms	8.2	[26]
Cd^2+^	2,4-D	0.060 mg/g a	*Alcaligenes eutrophus* JMP134	Field-scale bioreactors	8.2	[26]
Cd^2+^	2,4-DME	0.100 mg/L^a^	Indigenous community	Sediment (microcosm)	6.5	[16]
Cd^2+^	2,4-DME	0.629 mg/L a	Indigenous community	Aufwuchs (microcosm)	5.6	[16]
Cd^2+^	2,4-D	>3 mg/L a	*Alcaligenes eutrophus* JMP134	Mineral salts medium	6.0	[26]
Cd^2+^	2,4-D	24 mg/L a	*Alcaligenes eutrophus* JMP134	Mineral salts medium containing cadmium-resistant isolate	6.0	[26]
Cd^2+^	4-CP, 3-CB, 2,4-D	<25.3–50.6 mg/L a,b	*Alcaligenes* spp., *Pseudomonas* spp., *Moraxella* sp.	Tris-buffered minimal medium plates	7.0	[115]
Co^2+^	4-CP, 3-CB, 2,4-D	<13.3–1.330 mg/L a,b	*Alcaligenes* spp., *Pseudomonas* spp., *Moraxella* sp.	Tris-buffered minimal medium plates	7.0	[115]
Cr^3+^	2,4-DME	0.177 mg/L a	Indigenous community	Aufwuchs (microcosm)	6.1	[16]
Cr^6+^	4-CP, 3-CB, 2,4-D	<131 mg/L a,b	*Alcaligenes* spp., *Pseudomonas* spp., *Moraxella* sp.	Tris-buffered minimal medium plates	7.0	[115]
Hg^2+^	2,4-DME	0.002 mg/L a	Indigenous community	Aufwuchs (microcosm)	6.8	[16]
Hg^2+^	4-CP, 3-CB, 2,4-D	<45.2–226 mg/L a,b	*Alcaligenes* sp., *Pseudomonas* spp., *Moraxella* sp.	Tris-buffered minimal medium plates	7.0	[115]
Ni^2+^	4-CP, 3-CB, 2,4-D	5.18–10.3 mg/L a,b	*Alcaligenes* sp., *Pseudomonas* spp., *Moraxella* sp.	Tris-buffered minimal medium plates	7.0	[115]
Zn^2+^	2,4-DME	0.006 mg/L a	Indigenous community	Sediment (microcosm)	6.4	[16]
Zn^2+^	2,4-DME	0.041 mg/L a	Indigenous community	Aufwuchs (microcosm)	5.6	[16]
Zn^2+^	4-CP, 3-CB, 2,4-D	<29.5–736 mg/L a,b	*Alcaligenes* sp., *Pseudomonas* spp., *Moraxella* sp.	Tris-buffered minimal medium plates	7.0	[115]

3-CB, 3-chlorobenzoate; 4-CP, 4-chlorophenol; 2,4-D, 2,4-dichlorophenoxyacetic acid; DDT, 1,1,1-trichloro-2,2-bis(4- chlorophenyl)ethane; 2,4-DME, 2,4-dichloro-phenoxyacetic acid methyl ester; MTC, maximum total concentration; NR, not reported.

a Value represents total metal added to system;

b Value represents MIC calculated by multiplying MTC by a factor of 2.25 [10].

**Table 2:** Reported metal concentrations that cause inhibition of biodegradation of chlorinated organic contaminants under anaerobic conditions.

Metal	Organic	Lowest metal concentration	Microbe(s) studied	Environment	pH	Reference
Cd^2+^	TCA	0.01 mg/L a	Indigenous community	Laboratory soil microcosms containing rice paddy and bottomland hardwood soils	6.9–7.4	[95]
Cd^2+^	TCA	0.2 mg/L a	Indigenous community	Laboratory soil microcosms containing organic matter- rich soil	6.8	[95]
Cd^2+^	2-CP; 3-CB	0.5–1.0 mg/L b	Indigenous community	Aqueous sediment enriched in anaerobic growth medium	7.0	[15]
Cd^2+^	2-CP; 3-CP	20 mg/L b	Indigenous community	Sediment slurry	7.0	[73]
Cr ^6+^	2-CP; 3-CB	0.01–0.5 mg/L b	Indigenous community	Aqueous sediment enriched in anaerobic growth medium	7.0	[15]
Cu^2+^	2-CP, 3-CB	0.1–1.0 mg/L b	Indigenous community	Aqueous sediment enriched in anaerobic growth medium	7.0	[15]
Cu^2+^	2-CP; 3-CP	20 mg/L b	Indigenous community	Sediment slurry	7.0	[73]
Cr ^6+^	2-CP; 3-CP	20 mg/L b	Indigenous community	Sediment slurry	7.0	[73]
Pb^2+^	HCB	0.001 mg/g b	Indigenous community	Microcosms containing contaminated sediment	NR	[116]
Hg^2+^	2-CP; 3-CB	0.1–1.0 mg/L b	Indigenous community	Aqueous sediment enriched in anaerobic growth medium	7.0	[15]
Zn^2+^	PCP	2 mg/L b	Indigenous community	Anaerobic digester sludge in a liquid medium containing 0.6 mM phosphate	NR	[96]
Zn^2+^	PCP	8.6 mg/L b	Indigenous community	Anaerobic enrichment cultures in serum bottles	NR	[72]

3-CB, 3-chlorobenzoate; 2-CP, 2-chlorophenol; 3-CP, 3-chlorophenol; HCB, Hexachlorobenzene; PCP, Pentachlorophenol; NR, not reported; TCA, Trichloroaniline.

a Value represents solution-phase concentration of metal present in system;

b Value represents total metal added to system.
